# Systems-Aligned Precision Medicine—Building an Evidence Base for Individuals Within Complex Systems

**DOI:** 10.1001/jamahealthforum.2022.2334

**Published:** 2022-07-01

**Authors:** Anna R. Kahkoska, Nikki L. B. Freeman, Kristen Hassmiller Lich

**Affiliations:** Department of Nutrition, Gillings School of Global Public Health, University of North Carolina at Chapel Hill; Department of Biostatistics, Gillings School of Global Public Health, University of North Carolina at Chapel Hill; Department of Health Policy and Management, Gillings School of Global Public Health, University of North Carolina at Chapel Hill

Advances in data collection and storage, computing, and analytics, combined with a growing recognition of the clinical importance of heterogeneity among individuals, are producing new solutions to clinical problems and a paradigmatic shift in evidence generation: precision medicine. The tagline for precision medicine-right treatment, right patient, right time—distills the operating principle of tailoring treatment decisions to individuals’ unique characteristics and evolving health.^1-3^ While this goal is not new, what is novel is the growing capacity of analytic tools to generate data-driven strategies for increasing the precision of prevention and care.^2,4^ Put otherwise, the engine of empirical precision medicine is advanced analytics, and the fuel is patient-level data.

A statistical approach to precision medicine uses patient-level data and formalizes clinical decision-making into a model composed of patients’ features, key decision points, treatment options at those decision points, and primary outcomes to be optimized. What is remarkable about statistical precision medicine algorithms, known as *optimal dynamic treatment regimes*,^3^ is how they generate knowledge from data. Rather than relying on known or hypothesized relationships between interventions, health states, and outcomes, statistical and machine learning analytics can learn directly from the data how to tailor treatments optimally. The translational vision of precision medicine is to integrate optimal treatment recommendations into point-of-care tools that can offer real-time, tailored decision support.

Yet the question of what treatment works best for whom, to some degree, relies on broad and multilevel contextual factors. The clinical decision-making that happens as part of routine care, rather than in a research setting, is shaped by complexity at nearly every stage. Patient preferences, digitized workflows, billing and payment practices, and labor shortages can affect clinical decisions, and differences in resources, options for interdisciplinary care, and costs across care settings may restrict available treatment choices altogether. Furthermore, disparities in health care access, the social determinants of health (SDOH), and exposure to structural racism are formidable barriers that can prevent equitable access to optimal treatments, even if the underlying treatment rule could be statistically estimated. These factors extend far beyond the pared-down, formalized clinical decision-making models that underlie existing precision medicine algorithms.

An evidence base shaped by scientific methods that remove the variability in the actions, interactions, and environments of patients and clinicians alike, whether intentionally or unintentionally, will not address this real-world complexity. Health and health care decisions are shaped, and often constrained, by complex systems.^5^ While we tend to focus on formal systems, such as governmental or health care systems, a system can be any set of interconnected elements embedded in a structure that interact to determine outcomes of interest.^6^ What will work best for a given patient at a certain point in time is shaped by a web of patient factors (eg, symptoms, clinical history, preferences) in addition to clinician and institutional factors (eg, constrained time/resources) and societal factors (eg, access to care and differing SDOH-related health outcomes).

A new strategy for generating and evaluating evidence for precision medicine is needed—a strategy primed to translate data-driven individualized care strategies to be useful in complex systems. We call this strategy *systems-aligned precision medicine*, the goal of which is to deliver on the “right treatment, right patient, right time” tagline, but also to consider patients in their specific contexts.

Working at the systems level presents unique challenges. Problem-driven pragmatic solutions in precision medicine must thus start with defining the relevant system and its components. A systems-aligned approach then relies on broad and ongoing patient and other stakeholder engagement using structured participatory systems science methods to help untangle the clinical and nonclinical complexity relevant to a medical problem or population. This approach can illuminate diverse perspectives and priorities surrounding the potential interventions and identify constraints on decision-makers who represent the end users of future precision medicine decision support tools.^5,7^ A deep understanding of the relevant system also will facilitate the generation of complexity-aware precision medicine problem statements and subsets of discrete, solvable questions contained therein to increase the usefulness of precision medicine evidence in practice. Grounding precision medicine interventions in their specific systems will require new adjustments to traditional study designs, collection of varied process and outcome measures, and methodological triangulation to embed a system lens into evidence generation and evaluation.^8^ Additional quantitative modeling tools from the interdisciplinary field of systems science will be essential to design context-specific, data-driven clinical decision support algorithms.

The care of diabetes in older adults represents both the challenges and the opportunities for practicing a pipeline of systems-aligned precision medicine. One-quarter of US adults 65 years and older have diabetes, and their numbers are expected to increase as the population continues to age.^9,10^ Older adults with diabetes are a heterogenous population; thus, diabetes care must be carefully tailored to individuals’ medical, functional, and cognitive status.^10^ Moreover, the risk of hypoglycemia increases with age; severe events are associated with morbidity (eg, falls, fractures, hospital admissions) and mortality, so older adults often require adjustments of diabetes medications and self-management regimens to mitigate this risk over time. In the future, dynamic treatment regimens may offer valuable clinical decision support for diabetes care of older adults. These regimens could be designed to optimize the selection and dosing of medications, to identify the most appropriate and safe glycemic targets for patients, to match behavioral or technologic strategies to prevent hypoglycemia, and support adherence to broader care plans.

In addition to being tailored to older adults’ medical needs, however, precision medicine algorithms must also account for patients’ lived experiences and support networks. The system shaping diabetes outcomes for older adults includes many stakeholders (eg, patients, clinicians, payers, institutions), varied resources, evolving treatment options and best practices, and factors outside of the health care setting (eg, living situation, values, preferences, SDOH). Stakeholder-engaged system mapping with older adults and their caregivers may elucidate the interplay of diabetes self-care alongside the demands of managing other chronic diseases, constraints on treatment options regarding out-of-pocket costs and insurance rules, and challenges with navigating multiple specialty care experiences. To ensure that statistical models address the unique needs of different subpopulations, it is essential to include individuals who bring the greatest complexity, such as older adults with cognitive impairment or limited social support. The participatory methods applied with clinicians and health care institutions may further reveal the high-level patterns of how patients receive primary diabetes care, as well as the differing perspectives, roles, and responsibilities surrounding diabetes management in older adults across different settings. Understanding these dimensions of the larger system can elucidate opportunities to optimize patient-oriented dynamic treatment regimens that make sense when deployed across both primary care and specialty care settings and ensure the clinical decision support is utilitarian for diverse end users. Evaluation within and across settings can inform how differences in patient populations, systems-level resources, and clinical priorities shape whether, when, and how decision support can be used to tailor therapies for pragmatic and cost-effective health improvements among the population.

Precision medicine, to date, has been focused on individualized care decisions. A broader strategy of systems-aligned precision medicine is now needed, using patient data, stakeholder engagement, rigorous statistical methods, and an embedded awareness of complex systems to learn what is the right treatment for the right patient at the right time and in each patient’s unique context.
